# Effects of Isothermal Temperature on Dislocation Density in Bainite Transformation of 4140 Steel

**DOI:** 10.3390/ma15176066

**Published:** 2022-09-01

**Authors:** Jian Zhu, Gary Barber, Xichen Sun

**Affiliations:** 1 Tech Center, Stellantis North America, Auburn Hills, MI 48326, USA; 2 Department of Mechanical Engineering, Oakland University, Rochester, MI 48309, USA

**Keywords:** isothermal, upper and lower bainite, dislocation density, activation energy

## Abstract

To relate the bainitic microstructures to the mechanical properties of steel, the average dislocation density needs to be determined. Using X-ray diffraction and diffraction line broadening analysis, this research quantifies the average dislocation density in the four bainite phase matrices, (upper bainite, upper and lower bainite mixture, lower bainite, lower bainite and martensite mixture), which are transformed in a wide range of isothermal temperatures. The effects of isothermal temperatures on the average dislocation density are assessed for different thermal dynamic driving forces in terms of activation energy and cooling rate. It is found that as isothermal holding temperature is increased, the dislocation density in the bainite matrix decreases from 1.55 × 10^17^ to 8.33 × 10^15^ (m^−2^) due to the reduction in the plastic deformation in the austenite in the transformation. At the same time, the activation energy required decreases only after passing the martensite and lower bainite mixed phase. A new method for better estimating the average dislocation density in bainitic steel is also proposed.

## 1. Introduction

4140 steel is widely used in the applications of structures, tooling for manufacturing, and advanced automotive parts. 4140 steel with bainitic microstructure achieved through an isothermal process has enhanced mechanical properties such as high strength, good toughness, superior corrosion resistance, and extended fatigue life. One of the major contributors to these advantages is the high dislocation density [1,2,3] generated in the austenite-to-bainite transformation. Many dislocations exist at the austenite and bainite interface, in the bainite subunit laths, between the subunits, and even between the bainite sheaves [4]. The dislocations resist movement within the microstructures and hence strengthen the steels [1,2,3]. The higher the dislocation density, the higher the strength of the steel. The bainite transformation process is a process in which FCC structure is transformed into BCC structure. In these chemical and physical processes, the bainite phase matrix or an aggregate of phases, consisting predominately of ferrite platelets with a small amount of carbide and retained austenite [3], will be transformed. Bainitic ferrite plates usually nucleate on the austenite boundaries or on dislocations [4] within the parent austenite and grow into bainite sheaves, which are clusters of bainitic ferrite plates or subunits [1]. This process, which occurs inside the austenite, alters the austenite shape due to plastic deformation. There are many causes of higher dislocation density in bainitic steels, such as lower isothermal temperature, cold forming or ausforming of the steel before the bainite transmission, and lattice deformation, all of which can enhance the dislocation density [5,6,7,8]. From a microstructure standpoint, FCC austenite changes structure and shape to BCC bainitic ferrite during the phase transformation. The relaxation of the plastic deformation is accommodated in this shape deformation by the accompanying displacive or diffusion-less transformation, and results in the generation of high dislocation density [9,10] and dislocation debris [11,12,13]. Even from a diffusion-driven transformation standpoint, the completion of the transformation requires a “lattice invariant shear”, which creates and moves the dislocations during the bainite transformation [8].

Bainite and austenite interfaces are physically composed of dislocation arrays [1,3]. As the bainitic ferrite plates nucleate and grow, the dislocation arrays nucleate and develop. The dislocation density increases up to a peak level as the bainite transformation progresses [4]. The interface can be glissile or sessile. In the case of sessile, the dislocation arrays can only move by climbing or by a pile up [13]. During the transformation, carbon atoms partitioned from bainitic ferrites may be trapped, segregated, or redistributed to the dislocations in the vicinity of the ferrite austenite interface, which prevents the complete decarburization of the super saturated ferrite [14,15,16,17]. Local carbon enrichment slows down the bainite transformation kinetics since the carbon has a stabilization role in austenite [18]. The carbon enrichment will also produce plastic relaxation of the austenite, with fine bainite platelets generated [3]. The combined effect of the lower thickness of bainitic platelets, the thick bainite sheaves, and high dislocation density produces superior mechanical properties, such as high strength, ductility and toughness, and longer fatigue life [1,19,20,21,22,23,24,25]. The aggregate of bainite laths with nearly parallel slip systems between neighboring bainite laths (ALPS) is also one of the major contributors to these superior properties [24].

There are many methods to estimate the dislocation density in steels with bainitic microstructure. Using X-ray diffraction, it is done by analyzing the line broadening caused by bainite microstructure, Williamson and Smallman developed an equation to calculate the average dislocation density [26]. Since isothermal temperatures have a major influence on the bainite transformation, Takahashi and Bhadeshia developed an empirical relationship between bainite dislocation density and reaction temperature [1]. Garcia-Mateo and San Martin used high-resolution dilatometry [27,28] data to calculate the micro strain of the bainitic microstructure for estimating the dislocation density. Using Transmission Electron Microscopy (TEM) imaging and Convergent Beam Electron Diffraction (CBED), Williams and Cornide et al. also determined ways [29,30,31] to estimate bainite dislocation density. He and He et al. suggested that dislocation density in the bainitic phase matrix is distributed unevenly. The dislocation density on the bainite lath boundary is twice that on the lath center, and dislocations accumulate in the austenite that is between the laths [4]. Based on this observation of inhomogeneous distribution of dislocations and the Kocks–Mecking stress strain mode, He et al. developed a dislocation multiplication and annihilation model for the deformation behavior of bainitic steel. To effectively understand the steel’s mechanical properties such as strength and ductility, the average dislocation density of the steel needs to be calculated. For all the research studies mentioned above, almost all used the Williamson–Smallman equation and the XRD method to compare and validate their research, since it provides a relatively accurate estimation of the average dislocation density of the bainite phase matrix. This is also the reason why the Willamson–Smallman equation has been quoted in numerous dislocation density calculation related papers. 

The dislocation density is related to the bainite transformation, which can occur in a wide isothermal temperature range. In this wide range, upper bainite, upper and lower bainite mixture, lower bainite, and lower bainite and martensite mixtures are generated under different thermodynamic driving forces or undercooling [32]. The objective of this research is to calculate the average dislocation density of the four bainite phase matrices using X-ray diffraction data and a specific calculating method. This method uses a combination of the modified Williamson–Hall equations and Williamson–Smallman equations. This research will help to further understand the effects of isothermal temperature on the average dislocation density in all four groups of bainitic phase matrices in the 4140 steel, since dislocation density is a function of transformation temperature [2]. Quantifying the dislocation density achieved with slightly different microstructures can help direct the best applications of austempered 4140 steels.

## 2. Materials and Methods

### 2.1. Material

The as-received 4140 steel (by %wt: Cr 0.95, Mn 0.875, C 0.405, Si 0.225, Mo 0.20, S 0.04, P 0.35, Fe balance) has a hardness ranging from 25.6 HRC to 32 HRC; the steel also has a microstructure of proeutectoid phases embedded in a pearlite matrix.

### 2.2. Heat Treatment Experiment Procedures

**Figure 1:**
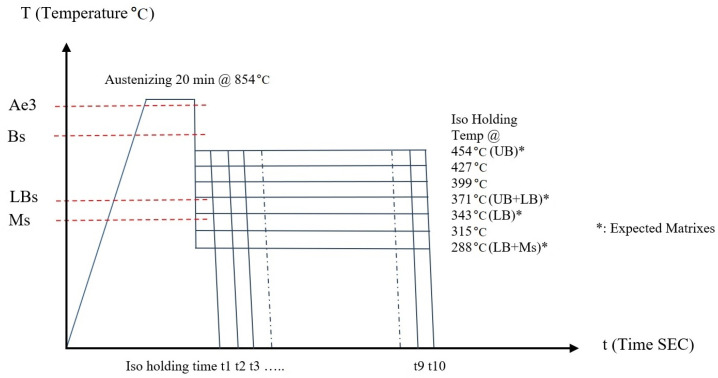
Isothermal processing experiment scheme.

Seven groups of samples (cylindrical shape, 20 mm in diameter with 5 mm thickness) of 4140 steel were prepared and all were austenitizied at 854 °C. The first group of the seven was processed at the isothermal holding temperature of 288 °C, the second at 316 °C, with 28 °C increments, respectively; the last group was processed at 454 °C. Figure 1 below is the experiment scheme.

With this wide isothermal temperature range, the following phase or mixed phases were expected to be generated: (a) upper bainite, (b) upper and lower bainite mixed, (c) lower bainite, (d) lower bainite and martensite mixture. For each group, ten samples for ten isothermal holding times were studied. After austenitizing for 20 min in a salt bath furnace, the steel samples were taken out of the salt bath and immediately placed into an isothermal holding furnace within 1 s; then, they were held in the holding furnace for 10, 30, 60, 90, 120, 150, 300, 600, 900, and 3600 s, ten holding times, (t1 to t10), respectively. Once the isothermal holding time was completed, the samples were dipped into water at room temperature to freeze the bainite transformation so the exact amount of bainite transformed could be determined through hardness measurement for kinetics calculations. 

The isothermal holding temperatures were selected from well below the bainite starting temperature (Bs) to a temperature below the martensite starting temperature (Ms); the martensite starting temperature was determined according to the TTT chart in the ASM Heat Treater’s Guide. The hardness measurements were conducted with a hardness tester for HRC reading. The metallographic samples were polished and etched with 2% nital. Microstructure observations were carried out under a light optical microscope, and microstructure images were taken with 500× magnification. 

X-ray diffraction was performed on the isothermal heat-treated samples at t10; X-ray line broadening profiles were measured. Line broadening analysis was used to determine the lattice strain (micro strain ε) and mosaic structure (crystallite size D). X-ray diffraction patterns were recorded for the 110 and 220 (Miller Indices) plane reflections of the samples. A graphite-monochromatic Cu–Ka radiation (wavelength = 0.17902 nm) was used. To accurately record the diffraction profile, a slow scanning speed of 0.5°/minute with 2θ divergence and receiving slits of 1° widths was used. 

## 3. Results and Analysis

The four groups of bainite phases were identified through kinetic curves, morphology, and activation energy analysis. X-ray diffraction peak profiles were measured for performing line broadening analysis.

### 3.1. Microstructures Observed

**Figure 2:**
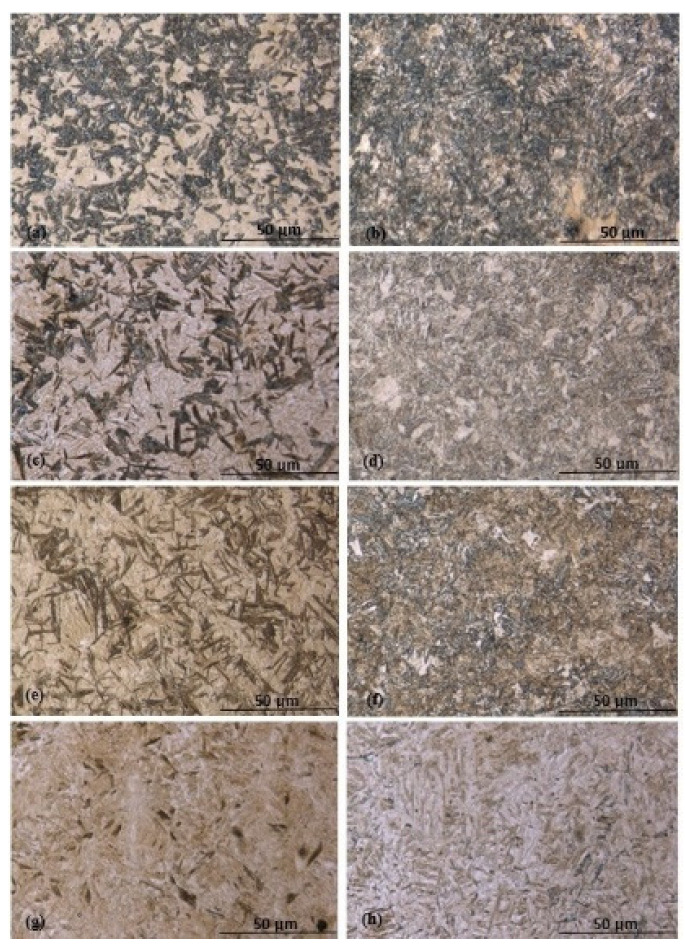
Various bainite observed in the experiments under LOM (500×): (**a**,**b**) UB at 427 °C, 30 s–900 s; (**c**,**d**) UB/LB mix at 371 °C, 30 s–600 s (**e**,**f**) LB at 343 °C, 60 s–600 s; (**g**,**h**) M/LB mix at 288 °C, 60 s–600 s.

By analyzing the images using optical microscopy and the bainite transformation kinetics curves obtained through hardness testing, the four groups of bainitic phases were identified. Upper bainite (UB) was produced between 454 °C and 399 °C in the experiments. Figure 2a–h shows the early stages of bainite microstructures during the bainite transformation under various isothermal holding times, when the bainite clusters are not too crowded and the microstructures are visually easy to see under LOM. Figure 2a,b show typical upper bainite microstructure. Upper bainite sheaves are short-fat and lath-like. Upper bainite and lower bainite mixtures were identified as the transformation products at an isothermal temperature of 371 °C; see Figure 2c,d. Lower bainite (LB), which consists of long-slim needle-like sheaves due to very fine carbide precipitation inside and between the bainitic ferrites, was produced when transformed at 343 °C; see Figure 2e,f. Lower bainite and martensite (M) mixed phases were identified as the transformation products at 316 °C and 288 °C; see Figure 2g,h.

### 3.2. X-ray Diffraction and Line Broadening Analysis

Williamson and Hall revealed that the X-ray peak breadths $B_{o\;}$ obtained using X-ray diffraction are a combination of the instrumental broadening and microstructure broadening. The breadth or line broadening can be attributed to both small particle size and strain broadening. The line broadening is fundamentally produced by dislocations or similar microstructure defects [33]. When considering the peak profiles as Gaussian profiles or using a Gaussian correction instead of Lorentz profiles or a Lorentz correction [32], the following equation can be used:

(1) $$B_{0}{}^{2}=\beta^{2}+b_{0}{}^{2},\;\mathrm{So}:\;\beta =\sqrt{B_{0}{}^{2}-b_{0}{}^{2}}$$

where *B*_0_ is the overall observed line broadening and *β* and *b*_0_ are the line broadening caused by microstructure and the instrument, respectively.

The breadths of the peak profiles of the isothermal heat-treated samples were used for overall line broadening analysis. For accuracy, the Full Width of Half Maximum (FWHM) peak profile [34] was measured as the overall line broadening; the <110> and <220> planes were scanned during X-ray diffraction.

The line broadening *β* can also be seen as the summation of the microstrain effect $\beta_{s}$ and the crystallite size effect $\beta_{D}$, according to modified Williamson and Hall, Fang, and Sun [31,32,35,36], where $\beta_{s}=4R\varepsilon \left(\tan\left(\theta \right)\right)$ and $\;\beta_{D}=\lambda R/D\left(\cos\left(\theta \right)\right)$. *R* is radius of goniometer; *λ* is X-ray wavelength (1.7902 Å); θ is Bragg angle, *D* and ε are crystallite size and microstrain of the isothermal heat-treated sample. In the experimental conditions used in this research, the Bragg angles for the <110> and <220> planes are calculated as $\theta_{110}$ = 26° and, $\theta_{220}$ = 62°. The overall line broadening β caused by the microstructure at the [*HKL*] (Miller indices) plane can also be expressed as:

(2) $$\beta_{\left[HKL\right]}=\beta_{s\left[HKL\right]}+\beta_{D\left[HKL\right]}=4R\varepsilon \left(\tan\left(\theta_{\left[HKL\right]}\right)\right)\;+\;\lambda R/D\left(\cos\left(\theta_{\left[HKL\right]}\right)\right)$$

with $\theta_{110}$ and $\theta_{220}$ known then as:

(3) $$\beta_{\left[110\right]}=4R\varepsilon \left(\tan\left(\theta_{\left[110\right]}\right)\right)+\;\lambda R/D\left(\cos\left(\theta_{\left[110\right]}\right)\right)$$

(4) $$\beta_{\left[220\right]}=4R\varepsilon \left(\tan\left(\theta_{\left[220\right]}\right)\right)+\;\lambda R/D\left(\cos\left(\theta_{\left[220\right]}\right)\right)$$

By resolving Equations (3) and (4), *D* and ε can be obtained.

### 3.3. Average Dislocation Density

Williamson and Smallman developed an equation to calculate average dislocation density when extensive polygonization or dislocation pileups were not considered [26]. They suggested that the average dislocation density *ρ* of a microstructure is a function of the microstrain and the crystallite size:

(5) $$\rho =\rho_{D}+\rho_{s}$$

where $\rho_{D}=3/D^{2}$ and $\rho_{s}=k\varepsilon^{2}/Fb^{2}$. Here, k=6π, F = 1, and *b* is the magnitude of the Burgers vector of the majority of the dislocations, calculated as *b* = 2.4823 Å.

Using the above equations and the data from X-ray line broadening analysis, the calculated results using experimental data are listed in Table 1:

For comparison, the average dislocation density was calculated using the Takahashi and Bhadeshia’s empirical equation using log (*ρ*) = C + 6880/T − 1780360/T^2^, using C = 1.2848 instead of 9.2848 [1] for more comparable results. Takaki et al. conducted research using Transmission Electron Microscopy (TEM) to estimate the dislocation density in cold rolled metal according to the Williamson and Hall equation and developed the following empirical equation [37] using the Gaussian correction *ρ* = 1.5 × 10^20^ε^2^ and with the Lorentz correction *ρ* = 1.9 × 10^20^ε^2^. These equations are used for reference purposes here and are applied under isothermal temperatures and microstrain. All analysis results are listed in Table 2. It can be seen that the X-ray Diffraction (XRD) methods using empirical equations yield similar results. The results found using the Takahashi equation [1] varies a small amount for the low transformation temperatures. The dislocation density calculated with X-ray diffraction in this study is slightly higher than that of most published results. This is similar to the findings by Martins [28]. The discrepancy is approximately a factor of ten; most likely, it is caused by the tetragonality [38] of the BCT unit cell, a variant of bainitic BCC due to trapped carbon solute atoms. The tetragonality will expand the line broadening when X-ray diffraction analysis is applied.

**Figure 3:**
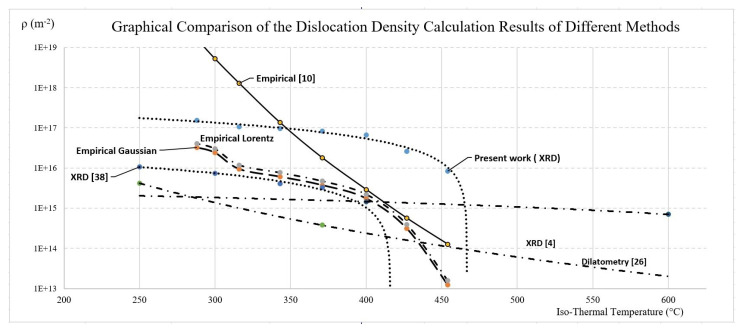
Graphical comparison of the dislocation calculations using different methods.

When analyzing the actual diffraction peak width, if only BCC lattice parameters are used, the derived estimated dislocation density can be overestimated. One way to improve the estimation would be to use BCT lattice parameters when calculating the Braggangles. However, not all BCC unit cells will show tetragonality. The alloy carbon content level, as well as the isothermal holding temperature, will heavily influence the percentage of BCC unit cells that would become BCT unit cells. It is very challenging to accurately find the percentages, and to weight and to aggregate the influence of the BCC and BCT unit cells on the expansion of the X-ray diffraction peak widths. Further research is needed to determine how to apply these results to the dislocation density estimation calculation. Figure 3 shows the graphical presentation of the data from Table 2. The resulting data trend line of the present work is parallel, but it is significantly offset from the XRD [22] results trend line. This may be due to the system error involved in the present work, which sets the upper bound of the dislocation density using XRD, while XRD [22] is the lower bound. The other results are within these bounds in the common isothermal temperature area, including the empirical equations.

### 3.4. Isothermal Temperature Effect on Dislocation Density & Hardness

**Figure 4:**
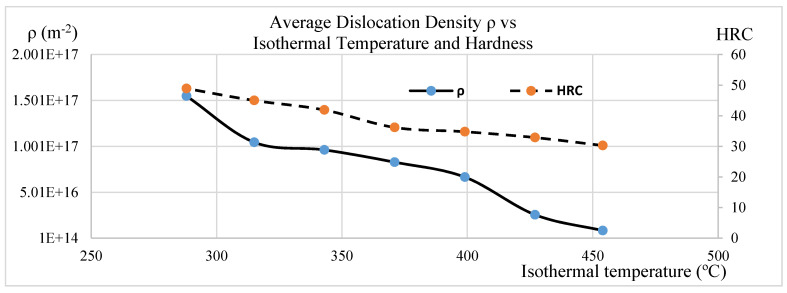
Isothermal temperature effect on dislocation density and hardness.

Figure 4 shows a plot of average dislocation density and hardness versus isothermal temperature. As the isothermal temperature increases from 288 °C to 454 °C, the dislocation density in the bainitic 4140 steel decreases from 1.55 × 10^17^ (m^−2^) to 8.44× 10^15^ (m^−2^). As the dislocation density decreases, the hardness decreases from 50 HRC to 30 HRC.

### 3.5. Bainite Phase Matrix Dislocation Density

**Figure 5:**
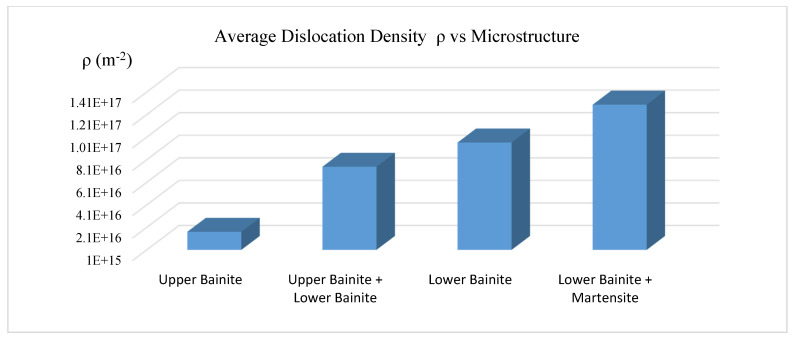
Dislocation density vs. microstructure.

Figure 5 shows that the bainite phase matrix transformed at lower isothermal temperature has higher average dislocation density.

### 3.6. Isothermal Temperature Effect on Crystallite Size D and Microstrain

**Figure 6:**
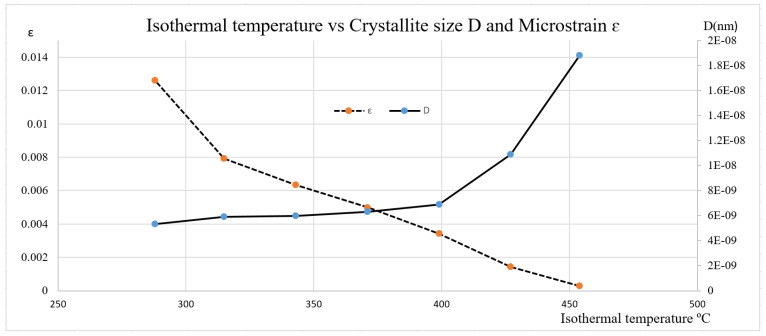
Isothermal temperature effect on crystallite size and microstrain.

Figure 6 shows the effect of isothermal temperature on the 4140 bainitic steel crystallite size and microstrain. As the isothermal temperature or bainite transformation temperature increases from 288 °C to 454 °C, the crystallite size increases from 5.31 × 10^9^ to 1.88 × 10^8^ (m^−2^), while the nouniform microstrain decreases from 0.0126 to 0.0003. This is why the microstructure, especially the bainitic ferrites, became coarse at the higher transformation temperatures.

### 3.7. Dislocation Density and Bainite Activation Energy

**Figure 7:**
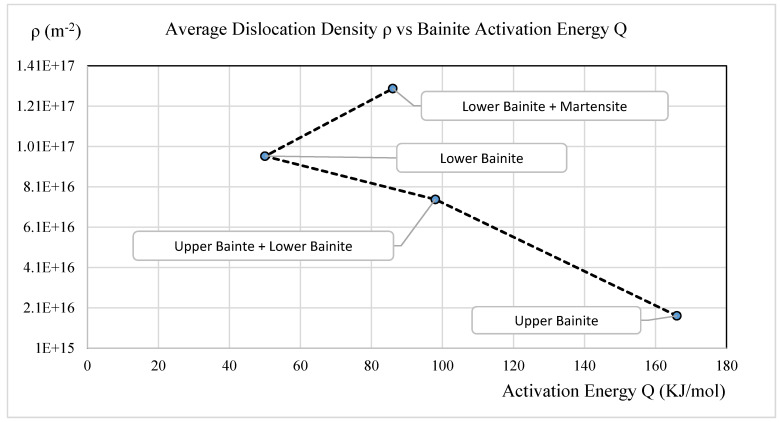
Average dislocation density vs. bainite activation energy.

The bainite transformation thermal dynamic driving force is directly proportional to the bainite activation energy, since free energy is needed to initiate the microstructure change in which dislocations will be generated and accumulated. This can be concluded mostly from Figure 7. At higher isothermal temperatures, upper bainite will be transformed; the activation energy needed is 167 KJ/mol [39] and the dislocation density average in the upper bainite region is 2.0 × 10^16^ (m^−2^); see Figure 7. As isothermal temperature is decreased to the lower bainite region, the activation energy needed decreases to 98 KJ/mol and the average dislocation density increases to 7.5 × 10^16^ (m^−2^). This trend continues when lower bainite is transfomed at even lower temperatures.

The activation energy needed decreases and the average dislocation density increases due to intense shape deformation. The trend is reversed when the isothermal temperature decreases to the lower bainite and martensite area. The activation energy needed is slightly higher due to the bainite transformation acceleration caused by pre-existing martensite, but the average dislocation density still follows the trend, increasing to 1.3 × 10^17^ (m^−2^). This can be explained by the high intricacy and higher density of the boundaries between the martensite, bainite, and austenite. The boundaries are composed of dislocation arrays, so the dislocation density also increases.

### 3.8. Average Dislocation Density vs. Cooling Rate

**Figure 8:**
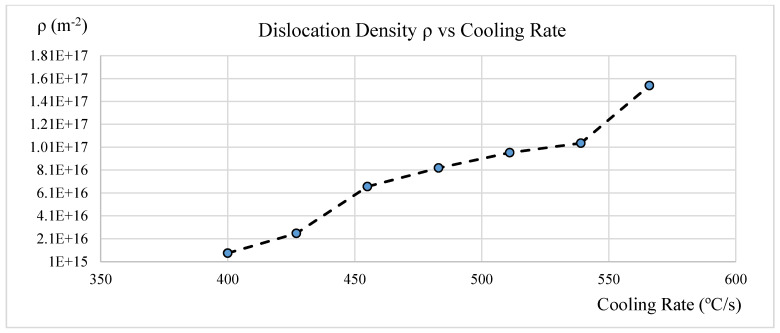
Cooling rate and average dislocation density.

Undercooling creates the thermal dynamic driving force of the phase transformation. The bainite phase transformation activation energy is proportional to the increase of the cooling rate. As the undercooling increases, the driving force increases, which drives the bainite phase transformation to lower isothermal temperatures. The greater the undercooling or the cooling rate is, the higher the dislocation density, which is also proportional to the steel hardness. This is shown in Figure 8.

## 4. Discussion

In this research, the impacts of calculated average dislocation density on many aspects of steel characteristics are considered; some discussion points arise below.

1. As isothermal temperature rises from 288 °C to 454 °C, the average dislocation density in the 4140 steel with bainitic microstructure decreases from 1.55 × 10^17^ (m^−2^) to 8.33 × 10^15^ (m^−2^). This is due to the reduction in the plastic deformation in the austenite during the transformation. As the dislocation density decreases, the hardness decreases from 50HRC to 30HRC. The dislocation density at the magnitude of +17 (m^−2^) level seems high, most likely caused by tetragonality, which can expand the line broadening.
2. As isothermal temperatures rise from 288 °C to 454 °C, four groups of bainite phase matrices will form, which are lower bainite and martensite mixture, lower bainite, lower bainite and upper bainite mixture, and upper bainite. The average dislocation density related to the four groups are 1.29 × 10^17^ (m^−2^), 9.62 × 10^16^ (m^−2^), 7.48 × 10^16^ (m^−2^), and 1.7 × 10^16^ (m^−2^), respectively.
3. The bainite transformation activation energies of all four microstructure groups were calculated and related to the dislocation density. As the activation energy decreases, the average dislocation density decreases only to the lower bainite phase. The trend reverses to higher free energy required with the lower bainite and martensite mixed phase due to the kinetic acceleration requirement of pre-existing martensite.
4. As the isothermal temperature rises, the microstructure crystallite size increases and the non-uniform micro strain decreases; in turn, the dislocation density decreases.
5. As isothermal temperatures decrease below the undercooling temperature of 454 °C and as the cooling rate increases from 400 °C/s to 566 °C/s, the thermal dynamic driving force of the bainite transformation increases. The average dislocation density in the bainitic phase matrix increases from 8.33 × 10^15^ (m^−2^) to 1.55 × 10^17^ (m^−2^).
6. The present work determined the average dislocation density. Due to the inhomogeneous dislocation distribution in the bainite phase matrix, the effect of differences between edge dislocations and screw dislocations was not considered.
7. The results of estimated average dislocation density obtained by this research are slightly higher than those of some published works, possibly due to the tetragonality of BCT unit cells. A remedy is proposed in the conclusions section.

## 5. Conclusions

Austempering or the isothermal process was carried out on 4140 steel over a wide temperature range. The heat-treated samples were studied using hardness testing as well as X-ray diffraction. Using X-ray line broadening analysis, the average dislocation densities were calculated and plotted for all seven isothermal temperatures studied in this research. The relationships between the average dislocation density and hardness, microstructure, crystallite size, microstrain, and activation energy, as well as cooling rate, were all characterized for the 4140 steel with the microstructure of the bainitic phase matrix.

To improve the average dislocation density calculation presented in this work, the impact of the tetragonality of BCT unit cells can be alleviated by using BCC lattice parameters with the additional use of BCT lattice parameters while applying the ratio of BCC to BCT’s influence on X-ray line broadening. This way, further research can be done to determine the percentage of BCC unit cells that become BCT at various carbon levels and isothermal holding temperatures. The results may show more realistic or lower average dislocation density comparing to this research work.

## Figures and Tables

**Table 1:** X-ray line broadening, cystallite size D, and microstrain ε.

Iso-Temp (°C)	$B_{o110}$ (mm)	$B_{o220}$ (mm)	$b_{o110}$ (mm)	$b_{o220}$ (mm)	$\beta_{\left[110\right]}$ (mm)	$\beta_{\left[220\right]}$ (mm)	*D* (nm)	ε
454	10.7	17.4	10.5	16.9	2.0591	4.1412	1.88 × 10^−8^	0.00028
427	11.2	18.9	10.5	16.9	3.8974	8.4616	1.09 × 10^−8^	0.00143
399	12.4	22.6	10.5	16.9	6.5962	15.0049	6.89 × 10^−9^	0.00341
371	13	24.8	10.5	16.9	7.6648	18.1502	6.31 × 10^−9^	0.00499
343	13.5	26.7	10.5	16.9	8.4852	20.6707	5.98 × 10^−9^	0.00635
316	13.9	28.5	10.5	16.9	9.1082	22.9486	5.92 × 10^−9^	0.00791
288	15.6	35.2	10.5	16.9	11.5373	30.8776	5.31 × 10^−9^	0.01261

**Table 2:** Comparison of the dislocation density ρ (m^−2^) calculated using different methods.

Isothermal Temp (°C)	Present Work (XRD)	Empirical Gaussian [37]	Empirical Lorentz [37]	Empirical Takahashi [1]	XRD [22]	Dilatometry [27]	XRD [4]
454	8.44 × 10^15^	1.24 × 10^13^	1.57 × 10^13^	1.27 × 10^14^			7.0 × 10^14^–1.46 × 10^15^ 400–600 °C
427	2.57 × 10^16^	3.10 × 10^14^	3.93 × 10^14^	5.64 × 10^14^			
400	6.67 × 10^16^	1.75 × 10^15^	2.21 × 10^15^	2.92 × 10^15^			
371	8.29 × 10^16^	3.74 × 10^15^	4.73 × 10^15^	1.79 × 10^16^	3.29 × 10^15^	3.77 × 10^14^	
343	9.62 × 10^16^	6.06 × 10^15^	7.67 × 10^15^	1.35 × 10^17^	4.11 × 10^15^		
316	1.05 × 10^17^	9.39 × 10^15^	1.18 × 10^16^	1.28 × 10^18^			
300		2.38 × 10^16^	3.02 × 10^16^	5.18 × 10^8^	7.43 × 10^15^		
288	1.55 × 10^17^	3.21 × 10^16^	4.07 × 10^16^	1.62 × 10^19^			
250					1.07 × 10^16^	4.2 × 10^15^	

## Data Availability

Not applicable.
